# Hypofractionated and conventionally fractionated radiotherapy for synchronous bilateral breast cancer: clinical and dosimetric outcomes

**DOI:** 10.1007/s00066-026-02558-5

**Published:** 2026-06-11

**Authors:** Bahar Cepni, Line Hoeltgen, David Neugebauer, Vania Batista, Nathalie Arians, Jakob Liermann, Lars Wessel, Maria Vinsensia, Semi Harrabi, Hanna Waldsperger, Thomas Tessonnier, Juliane Hörner-Rieber, Jürgen Debus, Eva Meixner

**Affiliations:** 1 Department of Radiation Oncology, Heidelberg University Hospital, Im Neuenheimer Feld 400, 69120 Heidelberg, Germany; 2 Heidelberg Institute of Radiation Oncology (HIRO), 69120 Heidelberg, Germany; 3 National Center for Tumor diseases (NCT), Heidelberg, Germany; 4 Heidelberg Ion Therapy Center (HIT), Im Neuenheimer Feld 450, 69120 Heidelberg, Germany; 5 German Cancer Research Center (DKFZ), Clinical Cooperation Unit Radiation Oncology, Im Neuenheimer Feld 280, 69120 Heidelberg, Germany; 6 Department of Radiation Oncology, University Hospital Düsseldorf, Düsseldorf, Germany

**Keywords:** Irradiation, Radiation dermatitis, Volumetric modulated arc therapy, Deep-inspiration breath hold, Tomotherapy, Toxicity

## Abstract

**Background:**

The irradiation of both breasts poses complex technical challenges. The overlapping treatment fields and large target volumes make dosimetry challenging, while data for hypofractionated regimens in this setting are limited.

**Methods:**

Bilateral breast cancer patients treated with moderately hypofractionated and conventionally fractionated radiotherapy were identified to evaluate dosimetric parameters, toxicity, and oncologic outcomes.

**Results:**

Seventy patients treated with a median dose of 50.4 Gy in 28 fractions or 40.05 Gy in 15 fractions using 3D-CRT, IMRT, VMAT, or tomotherapy were identified. With a median follow-up of 45.3 months, the 1‑ and 5‑year overall survival rates were 96.1% and 88.7%, and the local control rates were 100% and 92.5%, respectively. The median lung and heart doses were 8.4 and 4.0 Gy for conventional fractionation and 7.6 and 3.1 Gy for hypofractionation, respectively. With conventional fractionation, acute toxicity (≥ grade 3) occurred in 11% of patients (skin), and late toxicity occurred in 4% (skin and cardiac). With hypofractionation, acute toxicity (≥ grade 3) occurred in 8% (skin); no late grade ≥ 3 toxicity was observed. Even though late skin toxicity was more frequent with conventional fractionation, this did not reach statistical significance (*p* = 0.056). Regarding RT techniques, only the use of helical tomotherapy was associated with higher incidences of acute ≥ grade 3 skin toxicity (*p* = 0.029).

**Conclusion:**

Our long-term findings support the use of moderately hypofractionated RT with VMAT as a safe option for bilateral whole-breast irradiation for synchronous bilateral breast cancer, demonstrating favorable outcomes. However, prospective data are still needed, especially for nodal and bilateral chest wall irradiation involving implants.

## Introduction

With an estimated annual incidence of over 2 million new cases worldwide, breast cancer is the most common malignant disease in women [1]. Approximately 2% of breast cancer patients develop synchronous bilateral breast cancer [2]. When compared with unilateral breast cancer, there are conflicting data on the outcomes of synchronous and metachronous bilateral tumors, with worse treatment outcomes observed [3, 4]. Postoperative radiation therapy (RT) is considered the most effective measure for reducing the risk of locoregional recurrence [5, 6].

However, the irradiation of both breasts poses complex technical challenges. The overlapping treatment fields and large target volume make dosimetry challenging. This results in high integral body doses and increased doses to organs at risk (OAR), especially the lungs and the heart [7]. Furthermore, the risk of developing secondary cancer increases [8].

With advances in technology over recent years, there are various radiation therapy options for dose optimization to improve tumor coverage and organ sparing. While hypofractionated RT has become the preferred approach in early-stage disease [9], its role in more complex cases continues to be evaluated. The available data are limited and based mainly on theoretical planning analyses, without considering actual clinical effects and outcomes.

The aim of this retrospective study was to evaluate dosimetric parameters, toxicity, and oncologic outcomes in patients with bilateral breast cancer treated with moderately hypofractionated and conventionally fractionated RT.

## Methods

In this single-center retrospective review, we analyzed women treated with radiotherapy to both breasts for synchronous bilateral breast cancer between 2010 and 2025. The analysis was approved by the ethical review board (S-535/2025) and has therefore been performed in accordance with the ethical standards laid down in the 1964 Declaration of Helsinki and its later amendments. The requirement for written informed consent from each individual was waived by the appropriate institutional review board. All patients underwent upfront tumor resection. Only patients undergoing curative, postoperative treatment for synchronous bilateral tumors diagnosed within 3 months after diagnosis of the first tumor were included. Patients with prior RT to the chest or distant metastases were excluded. The recommendation for postoperative bilateral RT was confirmed in an interdisciplinary oncological conference that included radiation oncologists, gynecologists, oncologists, pathologists, and radiologists.

Radiotherapy planning was performed in a supine and arms-up position with an immobilization WingSTEP (IT V, Innsbruck, Austria) device. Target volume contouring was performed according to international guidelines from the European Society of Radiation Oncology (ESTRO) [10] using computer tomography images with a slice thickness of 3 mm. For RT treatments, photon-based techniques, including three-dimensional conformal RT (3D-CRT), intensity-modulated RT (IMRT), helical tomotherapy, and volumetric modulated arc therapy (VMAT) were utilized either in free breathing or with a deep-inspiration breath hold (DIBH) technique. Conventionally fractionated RT was applied with a total dose of 50–50.4 Gy in 25 to 28 fractions, while a total dose of 40–40.05 Gy in 15 to 16 fractions was used in hypofractionated regimens. In the case of higher local risk factors, a simultaneously integrated photon boost was applied using conventional fractionation (60–66.4 Gy) or hypofractionation (48 Gy). Alternatively, a sequential photon or electron boost (10–16 Gy) or an intraoperative RT boost (10 Gy) was used. The total planning target volume (PTV) was calculated as the cumulative sum of both the left- and right-sided PTVs. The RT plans aimed to deliver > 95% of the prescribed dose to cover ≥ 95% of the target. Dose constraints for adjacent OARs aimed to be in accordance with the QUANTEC recommendations [11]. Total dose, RT technique, and the use of a bolus were dictated by the patient and tumor status, anatomy, and at the discretion of the radiation oncologist.

For further comparison, doses were converted to an equivalent dose in 2‑Gy fractions (EQD2) using the linear quadratic model. An α/β ratio of 3 was assumed for normal tissue:

$$\begin{array}{c}\mathrm{EQD2\,Gy}=\begin{array}{c}\mathrm{fractional}\\\mathrm{dose}\end{array}\times\begin{array}{c}\mathrm{number\,of}\\\mathrm{fractions}\end{array}\\\times\left[\left(\begin{array}{c}\mathrm{fractional}\\\mathrm{dose}\end{array}+\alpha/\beta\right)/\left(\mathrm{2\,Gy}+\alpha/\beta\right)\right]\end{array}$$

However, to ensure clinical transparency, all original physical doses for the moderate hypofractionation schedules are also provided.

### Outcomes and toxicity

Follow-up included clinical examinations, referring physician notes, and imaging, all of which were used to evaluate the treatment response. Overall survival (OS) as well as locoregional (LC) and distant control (DC) were defined for each patient from the first day of RT until last contact or date of death. Acute and late toxicities were assessed according to the Common Toxicity Criteria in Adverse Events (version 5.0).

### Statistical analysis

Kaplan–Meier survival analysis and the log-rank test were performed to evaluate survival and further compare subgroups by applying the log-rank test or Cox regression. A *p*-value < 0.05 was defined as statistically significant. Uni- and multivariate Cox proportional hazard ratios (HRs) were used to assess the influence of cofactors. Patient and treatment characteristics were compared between hypofractionated and conventionally fractionated regimens using Mann–Whitney U and Pearson χ^2^ tests for continuous and categorical data, respectively. Statistical analysis was performed using SPSS statistical software (versions 25.0 and 28.0; IBM Corp., Armonk, NY, USA).

## Results

We identified a total of 70 patients (median of 59 years, range: 37–87 years) treated with radiotherapy for bilateral breast cancer between 2010 and 2025. Patient and treatment characteristics for both groups are shown in Table 1 and were comparable regarding demographic parameters, except for significant differences regarding younger age and more positive lymph nodes in the conventionally fractionated group.

**Table 1:** Patient and treatment characteristics.

Characteristic	Conventional fractionation	Hypofractionation	*p*-value
	Value (percentage or range)	Value (percentage or range)	
*Median age; years*	58 (37–87)	64 (45–84)	0.042
*Median BMI (kgm*^*-2*^*)*	25.4 (17.9–38.2)	26.6 (21.0–36.3)	0.762
*Median ECOG*	0 (0–1)	0 (0–2)	0.226
*Pathological tumor stage (right/left side)*			0.730/0.705
Tis	4 (9%)/6 (13%)	2 (8%)/1 (4%)	
T1	28 (62%)/26 (58%)	17 (68%)/17 (68%)	
T2	10 (22%)/7 (15%)	5 (20%)/5 (20%)	
T3	3 (7%)/2 (4%)	1 (4%)/0	
T4	0/4 (9%)	0/2 (8%)	
*Histology (right/left side)*			0.529/0.550
Invasive ductal carcinoma	29 (64%)/32 (71%)	18 (72%)/15 (60%)	
Invasive lobular carcinoma	11 (24%)/7 (16%)	3 (12%)/6 (24%)	
Ductal carcinoma in situ	4 (9%)/6 (13%)	2 (8%)/3 (12%)	
Other	1 (2%)/0	2 (8%)/1 (4%)	
*Lymph nodes*			0.025
Negative	26 (58%)	21 (84%)	
Positive	19 (42%)	4 (16%)	
*Hormone receptor*			0.669
Positive	44 (98%)	24 (96%)	
Negative	1 (2%)	1 (4%)	
*Her2neu*			0.502
Positive	6 (13%)	2 (8%)	
Negative	37 (82%)	22 (88%)	
Unknown	2 (4%)	1 (4%)	
*TNBC*			0.911
Yes	6 (13%)	2 (8%)	
No	39 (87%)	23 (92%)	
*Resection status*			0.453
Negative margin	45 (100%)	44 (98%)	
Positive margin	0	1 (2%)	

*BMI* body mass index, *ECOG* Eastern Cooperative Oncology Group Performance Status, *TNBC* triple-negative breast cancer

**Fig. 1:**
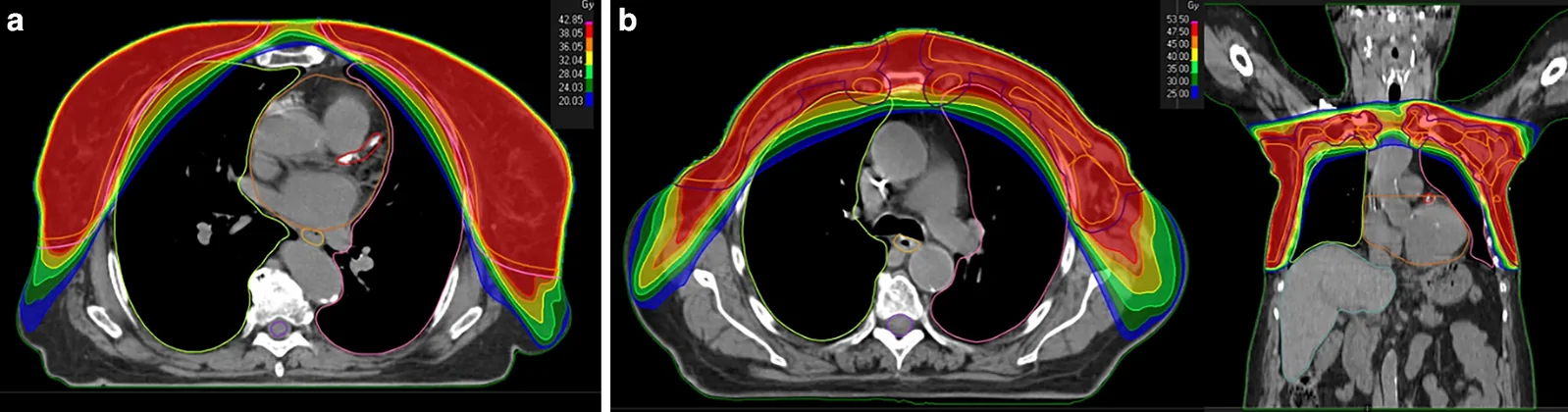
**a** Hypofractionated radiotherapy (RT) in a 85-year-old woman with a total dose of 40.05 Gy in 15 fractions. The planning target volume (PTV) was 2420 cm^3^ using free-breathing volumetric modulated arc therapy, with a mean equivalent dose in 2‑Gy fractions (EQD2) of 3.2 Gy to the heart and 9.8 Gy to the lungs. **b** Conventionally fractionated RT in a 75-year-old woman after bilateral mastectomy, with a total dose of 50 Gy in 25 fractions to the chest wall and regional nodes. The PTV was 1404 cm^3^, with a mean EQD2 of 4.1 Gy to the heart and 11.3 Gy to the lungs

A total of 45 patients were treated with conventionally fractionated RT with a median total dose of 50.4 Gy in 28 fractions. For the remaining 25 patients, moderately hypofractionated treatment regimens were applied, primarily VMAT (84%) with a median total dose of 40.05 Gy in 15 fractions. Figure 1 shows two patient examples of RT. A tumor bed boost was delivered in 52 patients (74.2%) using a simultaneously integrated (*n* = 25) or sequential (*n* = 27) boost with photons (*n* = 33), electrons (*n* = 15), or a mixed mode (*n* = 2); two sequential boosts were applied as intraoperative boosts. A tissue-equivalent bolus to improve dose coverage was used in five patients.

Significant treatment differences between the two groups were found: the number of mastectomies and axillary dissections, the use of 3D-CRT and tomotherapy, and nodal irradiation were significantly higher in the conventionally fractionated group. The PTV was significantly larger in the hypofractionated cohort (*p* = 0.021). This finding is largely explained by the higher prevalence of mastectomies within this subgroup, resulting in smaller target volumes compared to breast-conserving surgery (Table 2).

**Table 2:** Treatment characteristics.

Characteristic	Conventional fractionation	Hypofractionation	*p*-value
	Value (percentage or range)	Value (percentage or range)	
*Surgery*			0.016
Breast-conserving	38	24	
Mastectomy (implant)	7 (2)	1 (0)	
*Nodal surgery*			0.031
No	0	1	
Sentinel	31	21	
Axillary dissection	14	3	
*Systemic chemotherapy*			0.094
No	24	21	
Neoadjuvant	13	3	
Adjuvant	8	1	
*Endocrine therapy*	44	24	0.669
*Median dose, Gy (EQD2)*	50.4 (50–50.4; EQD2: 48.4)	40.05 (40–45; EQD2:45.4)	
*Median single dose, Gy*	1.8 (1.8–2)	2.67 (2.5–3)	
*Median number of fractions*	28 (25–28)	15 (15–16)	
*RT technique*			0.0001
3D-CRT	18	2	
IMRT	2	0	
Helical tomotherapy	13	1	
VMAT (DIBH)	12 (8)	22 (15)	
*Boost*	40	12	0.880
Simultaneous	19	6	
Sequential	21	6	
*Median PTV size, cm*^*3*^	1876 (791–4850)	2451 (967–4520)	0.021
*Target*			0.016
Breast	39	24	
Chest wall	6	1	
*RT of regional lymph node areas*	18	3	0.003
*Median PTV coverage, %*	95.6 (80–100)	94.4 (80–97.9)	–
*Median lung dose, Gy*	8.4 (3.6–17.3)	7.6 (2.9–12.1)	0.556
*Median lung dose V17Gy, %*	–	12.3 (7.0–23.6)	–
*Median lung dose V20Gy, %*	15.0 (0–30.0)	–	–
*Median heart dose, Gy*	4.0 (1.9–18.1)	3.1 (0.9–6.4)	0.264

*3D-CRT* three-dimensional conformal radiotherapy, *DIBH* deep-inspiration breath hold, *EQD2* equivalent dose in 2‑Gy fractions; *IMRT* intensity-modulated radiotherapy, *PTV* planning target volume, *VxGy* volume (%) that receives x Gy, *VMAT* volumetric modulated arc therapy

The median V20Gy lung dose (*p* = 0.006) and the median lung (*p* = 0.0001) and heart (*p* = 0.0001) doses in the conventionally fractionated group were significantly higher with tomotherapy compared to 3D-CRT and VMAT.

### Toxicity

Toxicity evaluation was performed for a median follow-up of 40.5 months (range: 0.7–101.0 months) for the hypofractionation cohort and for a median follow-up of 57.0 months (range: 1.7–170.4 months) for the conventional fractionation cohort; three patients were lost to follow-up and only included in the acute toxicity evaluation.

Detailed information on acute and late toxicity for both conventional and hypofractionation is presented in Tables 3 and 4.

**Table 3:** Acute and late toxicity graded according to the Common Terminology Criteria for Adverse Events (CTCAE, version 5.0)—conventional fractionation.

	*Number of events (%)*				
	*Grade 1*	*Grade 2*	*Grade 3*	*Grade 4*	*Grade 5*
*Acute adverse event*					
Skin disorders	21 (47%)	15 (33%)	5 (11%)	0	0
Fatigue	4 (9%)	0	0	0	0
Pain	5 (11%)	0	0	0	0
Lymphedema	3 (7%)	0	0	0	0
Dysphagia	0	0	0	0	0
Cardiac disorders	0	0	0	0	0
Nausea	0	0	0	0	0
Sensory neuropathy	0	0	0	0	0
Motor neuropathy	0	0	0	0	0
Respiratory disorders	0	0	0	0	0
Rib fracture	0	0	0	0	0
*Late adverse event*					
Skin disorders	12 (27%)	0	1 (2%)	1 (2%)	0
Fatigue	6 (13%)	1 (2%)	0	0	0
Pain	2 (4%)	0	0	0	0
Lymphedema	3 (7%)	0	0	0	0
Dysphagia	1 (2%)	0	0	0	0
Cardiac disorders	0	0	1 (2%)	0	0
Nausea	0	0	0	0	0
Sensory neuropathy	0	0	0	0	0
Motor neuropathy	0	0	0	0	0
Respiratory disorders	0	0	0	0	0
Rib fracture	0	0	0	0	0

**Table 4:** Acute and late toxicity graded according to Common Terminology Criteria for Adverse Events (CTCAE, version 5.0)—hypofractionation.

	*Number of events (%)*				
	*Grade 1*	*Grade 2*	*Grade 3*	*Grade 4*	*Grade 5*
*Acute adverse event*					
Skin disorders	19 (76%)	2 (8%)	2 (8%)	0	0
Fatigue	4 (16%)	0	0	0	0
Pain	1 (4%)	0	0	0	0
Lymphedema	0	0	0	0	0
Dysphagia	0	0	0	0	0
Cardiac disorders	0	0	0	0	0
Nausea	3 (12%)	0	0	0	0
Sensory neuropathy	0	0	0	0	0
Motor neuropathy	0	0	0	0	0
Respiratory disorders	0	0	0	0	0
Rib fracture	0	0	0	0	0
*Late adverse event*					
Skin disorders	5 (20%)	1 (4%)	0	0	0
Fatigue	6 (24%)	0	0	0	0
Pain	3 (12%)	0	0	0	0
Lymphedema	0	1 (4%)	0	0	0
Nausea	0	0	0	0	0
Dysphagia	0	0	0	0	0
Sensory neuropathy	0	0	0	0	0
Motor neuropathy	0	0	0	0	0
Respiratory disorders	0	0	0	0	0
Cardiac disorders	0	0	0	0	0
Rib fracture	0	0	0	0	0

Acute higher-grade toxicity was not significantly different (*p* = 0.718) between the two fractionation regimens and included grade 3 skin disorders in five patients (11%) with conventional fractionation and two patients (8%) with hypofractionation.

Conventionally fractionated RT resulted in late skin toxicity of grade 3 (*n* = 1; 2%) and grade 4 (*n* = 1; 2%; liponecrosis with fistula and surgical treatment). Additionally, one patient with grade 3 toxicity (*n* = 1; 2%,) experienced a cardiac disorder, which led to heart failure that necessitated multiple surgical treatments; this patient had a mean EQD2 heart dose of 11.8 Gy.

No higher-grade (≥ 3) late toxicity was observed in hypofractionated regimens. Even though late skin toxicity was observed more often in the conventionally fractionated group, this did not reach statistical significance (*p* = 0.056). All but one patient with conventionally fractionated treatment completed RT as planned. Acute grade 3 skin toxicity led to boost dose reduction in this patient. No acute or late sensory or motor neuropathy, rib fractures, or respiratory disorders/pneumonitis were observed.

Regarding RT techniques, only the use of helical tomotherapy was significantly associated with higher incidences of acute grade ≥ 3 skin toxicity (*p* = 0.029). Neither age (*p* = 0.301), body mass index (*p* = 0.800), patient performance status (ECOG; *p* = 0.220), type of surgery (*p* = 0.311), extent of lymph node dissection (*p* = 0.760), administration of systemic therapy (*p* = 0.331), or PTV volume (*p* = 0.116), nor the application of a boost (*p* = 0.898), significantly influenced the occurrence of toxicity.

### Survival outcomes

With a median follow-up of 45.3 months (range: 1.5–170.4 months) for the entire cohort, the 1‑, 2‑, and 5‑year overall survival rates were 96.1%, 93.6%, and 88.7%, respectively. Two in-breast recurrences were detected after 46.2 and 85.7 months, which resulted in 1‑, 2‑, and 5‑year local control rates of 100%, 100%, and 92.5%, respectively. Distant failure (*n* = 4 bone, *n* = 1 brain, *n* = 1 thoracic lymph nodes, *n* = 1 liver) was present in five patients, resulting in 1‑, 2‑, and 5‑year distant control rates of 96.2%, 93.6%, and 83.8%, respectively.

Only the tumor stage was significantly associated with DC (*p* = 0.041) in multivariate analysis. Histology, surgical approach, nodal status, application of chemotherapy, patient performance status, and BMI were not significantly associated with OS, LC, or DC. Treatment parameters, especially the fractionation regimens, were not significantly correlated to OS (*p* = 0.912), LC (*p* = 0.587), or DC (*p* = 0.914).

## Discussion

Our findings indicate that moderately hypofractionated RT for the treatment of synchronous bilateral breast cancer was well tolerated, with low rates of acute and late toxicities, aligning with previously reported effectiveness in terms of long-term oncologic outcomes.

Over the past decade, adoption of hypofractionated RT for postoperative treatment of breast cancer has significantly increased, supported by robust clinical evidence demonstrating that it is not inferior to conventional fractionation regarding local control, cosmetic outcomes, and toxicity profiles [9]. Landmark trials, such as the START trials [12], the Canadian trial [13], and the DBCG HYPO trial [14], have shown that moderately hypofractionated RT offers comparable efficacy to conventional regimens, with potential advantages in patient convenience, healthcare resource utilization, and cost-effectiveness. The transition from conventional to hypofractionated RT did not affect cosmetic outcomes, which were reported as good or excellent in 85% [14] and 69.8% [13] of patients after 5 and 10 years, respectively. However, despite its advantages and recommendation in the ESTRO guidelines for unilateral whole-breast, chest wall, nodal, and partial breast irradiation, several challenges remain regarding implementation [9]. Long-term data on late effects are still limited for certain subgroups, such as younger patients or those receiving immediate breast reconstruction. Finally, the RT treatment of both breasts, which was an exclusion criterion in the aforementioned studies, requires adjustments and data to maintain safety and efficacy.

The low local failure rates reported in our study are comparable with those in the literature, including studies by Offersen et al. [14] and Whelan et al. [13], who reported 9‑ and 10-year risks of local recurrence of 3.0% and 6.2%, respectively, when treating unilateral node-negative, early-stage breast cancer with hypofractionated RT. Ensuring a homogenous dose distribution and avoiding hotspots is even more complex in bilateral treatment and may require advanced planning techniques. The choice of radiation technique plays a critical role in achieving optimal tumor control while minimizing toxicity. Previous studies have already investigated a wide range of theoretical aspects of treatment planning without using real clinical data for outcome analysis.

Cho et al. [15] and Balaji et al. [16] identified modified hybrid plans as an optimal treatment technique for bilateral breast cancer and left regional nodes using a combination of VMAT and modified 3D-CRT plans for best PTV coverage and OAR protection. While dosimetric results were favorable, the actual patient treatment with hybrid plans and two isocenters may be prone to errors and is time-consuming. Kim et al. [7] evaluated dose–volume histograms and treatment efficiency for 3D-CRT, IMRT, and VMAT plans in 10 patients with bilateral treatment. While IMRT demonstrated superior sparing of OAR and better target coverage than 3D-CRT and VMAT, more favorable treatment times and efficiency were reached with the VMAT technique; VMAT was also considered superior to other forms of RT in other studies [17–20] and was the most commonly utilized technique according to a Canadian survey [21]. Furthermore, respondents prioritized target volume coverage (70%), followed by normal tissue sparing (25%) [21].

Nicolini et al. demonstrated dosimetric advantages of using an SIB with volumetric modulated arc therapy instead of fixed-field IMRT [22]. Huang et al. [23] compared fixed-jaw and tangential partial IMRT with single or two isocenters in 12 patients. They reported that all plans were of high quality and feasible, while fixed-jaw IMRT with a single isocenter was their preferred technique for local hotspot control in the PTV and for reducing OAR exposure at low dose levels.

While planning studies have presented proton beam therapy [24–27] as superior for sparing cardiac structures, its application presents clinical challenges in terms of guaranteeing robustness and accurate image guidance during RT. Furthermore, the use of particle therapy is not yet standardized, and the high cost and limited availability of facilities make widespread implementation challenging.

Cheng et al. [28] simulated a planning comparison study in 10 patients to compare dosimetric features of conventionally fractionated helical tomotherapy, VMAT, IMRT, and the tangential field-in-field technique. Due to lower doses to the heart and lungs, they recommended the use of helical tomotherapy as the most favorable technique for sparing OAR. Other studies identified helical tomotherapy with an SIB as a technically advantageous approach for the treatment of bilateral breasts with superior OAR sparing [29] and high safety in 21 women treated with conventionally fractionated RT [30]. Conversely, in our cohort, the use of helical tomotherapy was significantly associated with the presence of higher-grade (≥ 3) skin toxicity in the overall cohort. This also reflects the results of the study by Kaidar-Person et al. [31], who observed notable toxicity associated with tomotherapy [31], with relatively high complications in a cohort of nine patients who received a median cardiac dose of up to 20 Gy.

In our cohort, 88% of the women treated with hypofractionation received VMAT. In 68% of them, DIBH was technically and anatomically feasible. The use of such modern RT techniques may have contributed to the favorable outcomes and toxicity profiles observed in our cohort. Comparably, Tamilarasu et al. [32] and Volpe et al. [33] investigated the usefulness of DIBH VMAT compared to normal-breathing 3D-CRT and VMAT in theoretical planning studies and achieved better OAR sparing in conventionally fractionated DIBH plans.

The lungs and heart are the most critical adjacent organs at risk during RT, necessitating careful dose optimization. Treatment-related factors that influence the incidence and risk of heart disease [34] and pneumonitis are reported to be especially highly dependent on the mean doses to the heart and lungs [35–38]. In our cohort, the median EQD2 heart dose, lung dose, and lung V20Gy/V17Gy were approximately 4 Gy, 8.4 Gy, and 15.0% in the conventionally fractionated group and 3.5 Gy, 8.6 Gy, and 12.3% in the hypofractionated group, respectively. These values are lower than the doses reported in previous studies for the heart (Dmean of 3.7–22.3 Gy) [39–42] and the lungs (Dmean of 7.7–18.7 Gy, V20Gy of 15.3–29%) [31, 40–42]. However, the range of dose values was considerably high and largely determined by the RT technique applied.

While differences in planning between centers can occur due to variations in equipment, software, treatment protocols, and individual experiences and guidelines, our results confirm that theoretical planning models must be validated against clinical outcomes to confirm their effectiveness and ensure their practical accuracy.

However, real-world clinical data are limited. Across five studies evaluating conventionally fractionated RT for bilateral breast cancer, patient numbers ranged from 4 to 54 [41–45]. Most studies reported favorable toxicity profiles, with no grade ≥ 3 adverse events in most cohorts. One study with 54 patients [41] reported limited grade 3 toxicities, including acute skin reactions (3.7%) and late fibrosis (2%). Techniques such as VMAT and field-in-field IMRT achieved satisfactory target coverage with low OAR exposure (*n* = 7) [42]. Only one study with bilateral regional nodal irradiation [45] (*n* = 12) demonstrated a higher toxicity rate, with 8% grade 3 dyspnea. Overall, conventionally fractionated radiotherapy demonstrated acceptable safety and dosimetric profiles when modern techniques were applied. The clinical evidence on conventionally fractionated RT is still limited, with even fewer clinical data available for hypofractionation.

A study by McKenzie et al. comprises the largest cohort to date presenting hypofractionated bilateral RT [46]. These authors reported the oncologic and dosimetric results of 88 patients, 37 of whom were treated within a hypofractionated regimen of 42.56 Gy in 16 fractions. Although the study included a very large number of patients, its oncologic outcomes were suboptimal, with 10% local recurrences and a high risk of poor cardiac and pulmonary outcomes. This limitation is likely attributable to the exclusive use of 3D-CRT. Almost 14% and 42% experienced cardiac and pulmonary events after radiation, respectively. The median heart and lung doses were significantly associated with their occurrence. Moreover, 24% developed symptomatic lymphedema.

With the implementation of modern RT techniques such as VMAT or deep-inspiration breath hold, significantly reduced cardiac and pulmonary doses can be achieved. Gadea et al. treated a cohort of 20 women with bilateral breast cancer with hypofractionated VMAT RT and reported no higher-grade (≥ 3) toxicity, albeit with a short median follow-up of only 9 months [39].

Major limitations of the present study include the retrospective, single-center nature of the review, which limits the interpretation of the data. Obviously, moderate hypofractionation was introduced later in the study period, which is inherently reflected in the significantly shorter median follow-up for this subgroup compared to patients receiving conventional fractionation. Consequently, the potential for underestimating late-onset chronic adverse events in the hypofractionated cohort must be acknowledged, as the observation window may not yet capture the full spectrum of long-term toxicity.

Furthermore, a strong correlation exists between the era of treatment, the delivery technique, and the fractionation scheme; specifically, 3D conformal radiation therapy and tomotherapy were utilized almost exclusively in the conventional fractionation group. This collinearity limits our ability to assess the impact of fractionation independently of the technological advancements in radiation delivery. While our data reflect the real-world transition toward the modern standard of care, these confounding temporal factors should be considered when interpreting the comparative outcomes and toxicity profiles.

Due to the variety of radiation techniques applied over two decades, further contributions to the sparse literature are greatly needed. Only a limited number of the hypofractionated group received nodal irradiation, which limits the reliability of the results for this subgroup. Furthermore, this setting did not allow for a structured assessment of cosmetic outcomes. The interaction between hypofractionated RT and newer systemic therapies also remains an area of ongoing research. Nevertheless, our cohort provides long-term outcomes of over 3 years for hypofractionated and nearly 5 years for conventionally fractionated RT in a challenging patient subgroup, contributing to management optimization for bilateral breast cancer. Our data provide further clarity regarding the safety and efficacy of hypofractionated VMAT in this setting.

## Conclusion

Our long-term findings support moderately hypofractionated RT using volumetric modulated arc therapy as a safe option for bilateral whole-breast irradiation of synchronous bilateral breast cancer, demonstrating favorable outcomes. The choice of a modern radiotherapy technique is crucial for achieving optimal outcomes. Further research involving a larger patient cohort and data for bilateral nodal, chest wall, and implant irradiation are required to verify these results.

## Data Availability

All data supporting the findings of this work are included in the article.

## Abbreviations

3D-CRT: Three-dimensional conformal radiotherapy
DC: Distant control
DIBH: Deep-inspiration breath hold
EQD2: Equivalent dose in 2‑Gy fractions
IMRT: Intensity-modulated radiotherapy
LC: Local control
OAR: Organs at risk
OS: Overall survival
RT: Radiotherapy
VMAT: Volumetric modulated arc therapy
